# Cryo-EM reveals binding of linoleic acid to SARS-CoV-2 spike glycoprotein, suggesting an antiviral treatment strategy

**DOI:** 10.1107/S2059798323000049

**Published:** 2023-01-20

**Authors:** Christine Toelzer, Kapil Gupta, Imre Berger, Christiane Schaffitzel

**Affiliations:** a School of Biochemistry, University of Bristol, 1 Tankard’s Close, Bristol BS8 1TD, United Kingdom; b Bristol Synthetic Biology Centre: BrisSynBio, 24 Tyndall Avenue, Bristol BS8 1TQ, United Kingdom; c Imophoron Ltd, St Philips Central, Albert Road, Bristol BS2 0XJ, United Kingdom; d Max Planck Bristol Centre for Minimal Biology, Cantock’s Close, Bristol BS8 1TS, United Kingdom; Imperial College London, United Kingdom

**Keywords:** cryogenic electron microscopy, betacoronaviruses, SARS-CoV-2, spike protein, free fatty acids, linoleic acid, phospholipase A_2_, drug design

## Abstract

The COVID-19 pandemic and concomitant lockdowns presented a global health challenge and triggered unprecedented research efforts to elucidate the molecular mechanisms and pathogenicity of SARS-CoV-2. The spike glyco­protein decorating the surface of SARS-CoV-2 virions is a prime target for vaccine development, antibody therapy and serology as it binds the host cell receptor and is central for viral cell entry. The electron cryo-microscopy structure of the spike protein revealed a hydrophobic pocket in the receptor-binding domain that is occupied by an essential fatty acid, linoleic acid (LA). The LA-bound spike protein adopts a non-infectious locked conformation which is more stable than the infectious form and shields important immunogenic epitopes. Here, the impact of LA binding on viral infectivity and replication, and the evolutionary conservation of the pocket in other highly pathogenic coronaviruses, including SARS-CoV-2 variants of concern (VOCs), are reviewed. The importance of LA metabolic products, the eicosanoids, in regulating the human immune response and inflammation is highlighted. Lipid and fatty-acid binding to a hydrophobic pocket in proteins on the virion surface appears to be a broader strategy employed by viruses, including picornaviruses and Zika virus. Ligand binding stabilizes their protein structure and assembly, and downregulates infectivity. In the case of rhinoviruses, this has been exploited to develop small-molecule antiviral drugs that bind to the hydrophobic pocket. The results suggest a COVID-19 antiviral treatment based on the LA-binding pocket.

## Abstract 2

SARS-CoV-2 spike protein comprises a conserved hydrophobic pocket that binds linoleic acid (LA). LA supplementation interferes with viral entry into the cell and replication, and thus could be used as an antiviral.

## 1. Introduction

The spike glycoprotein decorates the surface of severe acute respiratory syndrome coronavirus 2 (SARS-CoV-2). Spike makes the first contact with the receptor on the host cell, human angiotensin-converting enzyme 2 (ACE2), and mediates infection by virus–host cell membrane fusion (Hoffmann, Kleine-Weber, Schroeder *et al.*, 2020 ▸; Letko *et al.*, 2020 ▸; Walls *et al.*, 2020 ▸). For this reason, spike is of particular interest for vaccine development and the generation of monoclonal antibodies to combat viral infection. Moreover, spike is used as a target for antivirals and in serology tests to test for the presence of specific antibodies. When the COVID-19 pandemic started, the first cryo-EM structures of SARS-CoV-2 spike glycoprotein became available very quickly, in February and April 2020 (Wrapp *et al.*, 2020 ▸; Walls *et al.*, 2020 ▸). This pioneering work demonstrated that starting from a sequence, it is now possible to produce a sample and solve its cryo-EM structure within merely weeks. The SARS-CoV-2 spike trimer showed an architecture that was very similar to previous spike protein structures of the *Betacoronavirus* genus, for example SARS-CoV (which caused an outbreak in 2002) and Middle East respiratory syndrome coronavirus (MERS-CoV), which was first identified in 2012 (Yuan *et al.*, 2017 ▸; Park *et al.*, 2019 ▸).

**Figure 1:**
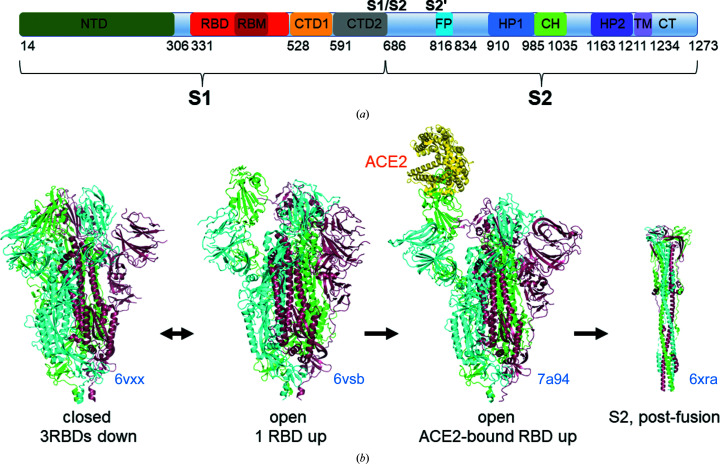
SARS-CoV-2 spike protein structure and interaction with the ACE2 receptor. (*a*) Schematic of the full-length spike protein. The S1/S2 furin-cleavage site and the TMPRSS2 S2′ cleavage site are indicated in bold. Residues at the domain boundaries are indicated. NTD, N-terminal domain; RBD, receptor-binding domain; RBM, receptor-binding motif; CTD1/CTD2, carboxy-terminal domains 1 and 2; FP, fusion peptide; HP, heptad repeat; CH, central helix; TM, transmembrane helix; CT, C-terminal tail. (*b*) Spike-protein structures illustrating the conformational changes that are required for receptor binding and cell entry. The closed state of the spike trimer with three RBDs down (PDB entry 6vxx; Walls *et al.*, 2020 ▸) is incompatible with receptor binding. The closed spike conformation is in equilibrium with the open state, with one or several RBDs up (PDB entry 6vsb; Wrapp *et al.*, 2020 ▸). ACE2 can be bound by RBDs in the up position (PDB entry 7a94; Benton *et al.*, 2020 ▸). ACE2 binding facilitates S2′ cleavage, dissociation of S1 and a drastic conformational change to the post-fusion state of S2. The post-fusion state comprises a long three-stranded coiled coil which brings the fusion peptide close to the host cell membrane, facilitating membrane fusion and viral entry (PDB entry 6xra; Cai *et al.*, 2020 ▸).

Spike protein forms a homotrimer. Host furin-like proteases cleave spike monomers into two fragments, S1 and S2, which remain associated (Hoffmann, Kleine-Weber & Pöhlmann, 2020 ▸; Jackson *et al.*, 2022 ▸; Fig. 1 ▸ *a*). The furin-cleavage site in spike is key to the increased pathogenicity of SARS-CoV-2 (Hoffmann, Kleine-Weber & Pöhlmann, 2020 ▸; Peacock *et al.*, 2021 ▸; Johnson *et al.*, 2021 ▸). S1 contains an N-terminal domain (NTD) and a receptor-binding domain (RBD) responsible for interaction with the ACE2 receptor, followed by C-terminal domains CTD1 and CTD2 (Hoffmann, Kleine-Weber, Schroeder *et al.*, 2020 ▸; Fig. 1 ▸ *a*). S2 comprises the membrane-fusion machinery, including the fusion peptide (FP), the fusion peptide-proximal region, heptad repeat (HP) 1, a central helix region (CH), HP2, a transmembrane anchor and a cytoplasmic C-terminal tail (Hoffmann, Kleine-Weber, Schroeder *et al.*, 2020 ▸; Jackson *et al.*, 2022 ▸; Fig. 1 ▸ *a*). The central helices of spike form a coiled-coil trimeric core. When spike interacts with ACE2, the S2 core and a second proteolytic site (S2′) become exposed (Benton *et al.*, 2020 ▸). Cleavage of S2′ by human transmembrane protease serine 2 (TMPRSS2; Takeda, 2022 ▸) and dissociation of S1 monomers release the fusion peptide and thus activate S2 for membrane fusion. To switch from the pre-fusion to the post-fusion conformation, the S2 trimer undergoes major conformational rearrangements (Fig. 1 ▸ *b*) leading to an elongated coiled-coil structure formed by HP1, the central helix and HP2 which positions the N-terminal fusion peptide close to the host cell membrane (Cai *et al.*, 2020 ▸).

The polybasic furin-cleavage site with amino-acid sequence RRAR is a transmission-enhancing feature acquired by SARS-CoV-2 that is not present in SARS-CoV (Hoffmann, Kleine-Weber & Pöhlmann, 2020 ▸). A furin-cleavage site has also been identified in MERS-CoV spike and is found in the cell-entry proteins of other highly pathogenic viruses such as avian influenza viruses, Ebola virus and HIV-1 (Jackson *et al.*, 2022 ▸). Deletion or mutation of the furin-cleavage site interferes with viral infection of lung cells and primary human airway epithelial cells (Peacock *et al.*, 2021 ▸; Johnson *et al.*, 2021 ▸). However, in patients infected with SARS-CoV-2, spike-protein variants with a deleted furin-cleavage site have been identified as a subpopulation allowing the virus to infect other cell types, including epithelial cells (Peacock *et al.*, 2021 ▸; Gupta *et al.*, 2022 ▸; Pohl *et al.*, 2021 ▸; Ogando *et al.*, 2020 ▸; Sasaki *et al.*, 2021 ▸; Klimstra *et al.*, 2020 ▸). Interestingly, more recent SARS-CoV-2 variants of concern (VOCs) have acquired even more basic cleavage sites (HRRAR in Alpha and Omicron spike proteins and RRRAR in Delta spike proteins), resulting in a more complete cleavage at the furin site, contributing to the increased transmissibility of these VOCs (Wrobel *et al.*, 2022 ▸).

To interact with the ACE2 receptor, the RBDs of spike need to adopt an ‘up’ conformation in which the receptor-binding motif (RBM) of the RBD is accessible (Fig. 1 ▸ *b*). In the closed conformation of spike the RBDs are all ‘down’, resulting in threefold symmetry. Importantly, the closed conformation of spike is considered to be a non-infectious form because the RBM is inaccessible. ACE2 interacts with spike in open states with one, two or all three RBDs pointing ‘up’ (Fig. 1 ▸ *b*), and it has been suggested that ACE2 interaction enhances the opening of the RBDs (Benton *et al.*, 2020 ▸).

## 2. Cryo-EM of SARS-CoV-2 spike glycoprotein

**Figure 2:**
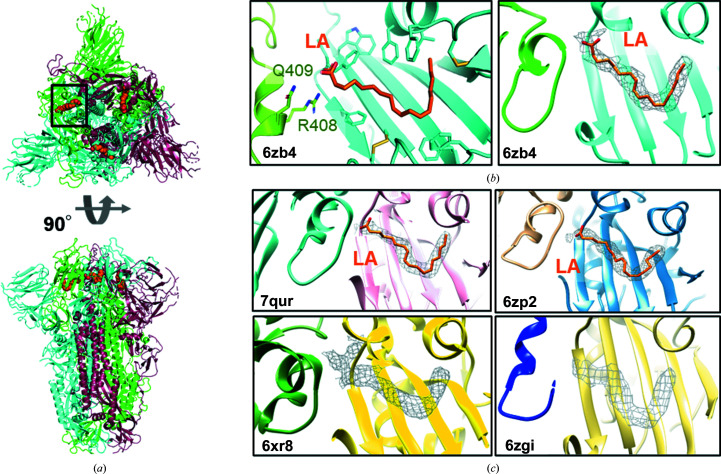
Structure of the spike glycoprotein with LA bound to the hydrophobic pocket. (*a*) The spike structure is shown in a cartoon representation in a top view (above) and a side view (below). Bound LA is shown as orange spheres. The black box highlights an LA-binding pocket. (*b*) The LA-binding pocket formed by adjacent RBDs (PDB entry 6zb4; Toelzer *et al.*, 2020 ▸). Left: the hydrophobic pocket (cyan RBD) is lined mostly by phenylalanines. The carboxyl head group of LA (orange) is coordinated by Arg408 and by Gln409 from the neighbouring RBD (green RBD). Right: the corresponding tube-shaped EM density for LA is shown (mesh). (*c*) Example structures of LA occupying the hydrophobic pocket of spike. A 2.27 Å resolution structure of LA-bound spike produced in insect cells (PDB entry 7qur; Buchanan *et al.*, 2022 ▸). Spike produced in mammalian cells with LA in the pocket (PDB entry 6zp2; Xiong *et al.*, 2020 ▸). Unassigned density in the full spike protein structure (PDB entry 6xr8; Cai *et al.*, 2020 ▸) and in furin-cleaved spike produced in mammalian cells (PDB entry 6zgi; Wrobel *et al.*, 2020 ▸).

The production of spike proteins from betacoronaviruses initially relied on expression of the ectodomain (the soluble extracellular spike domains) in mammalian cells and stabilization of the spike trimer by a trimerization domain (Kirchdoerfer *et al.*, 2016 ▸; Walls *et al.*, 2016 ▸; Amanat *et al.*, 2020 ▸). Spike production in the MultiBac insect-cell expression system (Fitzgerald *et al.*, 2006 ▸) was quality-controlled by cryo-EM (Toelzer *et al.*, 2020 ▸) and subsequently used for vaccine development and serology (Avolio *et al.*, 2021 ▸; Goenka *et al.*, 2021 ▸). In contrast to the previously reported structures (Wrapp *et al.*, 2020 ▸; Walls *et al.*, 2020 ▸), cryo-EM of insect-cell-produced spike showed a majority of spike particles in a closed conformation (∼70%); the corresponding *C*3-symmetrized structure reached a resolution of 2.85 Å. Surprisingly, the RBDs moved closer to each other compared with available atomic models. In a pocket of the RBD, additional tube-shaped density was identified which could not be attributed to protein (Fig. 2 ▸). The surrounding highly hydrophobic environment of the RBD, consisting mainly of phenylalanines, indicated a hydrophobic molecule. Comparison with published fatty acid–protein structures suggested that the length and shape of the density agreed with linoleic acid (LA), an unsaturated C_18_ fatty acid with two double bonds. Using hydrophobic interaction liquid chromatography–electrospray ionization mass spectrometry (HILIC-MS) and LC-MS-MS, the ligand in the RBD was confirmed to be LA (Toelzer *et al.*, 2020 ▸; Gupta *et al.*, 2022 ▸). Notably, only LA and no other fatty acids were identified by mass spectrometry (Toelzer *et al.*, 2020 ▸).

LA binding to the hydrophobic pocket in the RBD changes the closed conformation of the spike trimer. LA stabilizes the ‘down’ conformation of the RBD and induces a ‘locked’ conformation in which the RBDs are closer to each other compared with the closed LA-free conformation (Toelzer *et al.*, 2020 ▸). This conformational change to ‘locked’ allows the formation of a bipartite binding pocket for LA, in which the hydrophobic tail is bound to the pocket in one RBD, and the carboxyl headgroup of LA interacts via a salt bridge with an arginine (Arg408) and by hydrogen bonding to a glutamine residue (Gln409) of the neighbouring RBD (Figs. 2 ▸ *a* and 2 ▸ *b*). Notably, the RBM is completely structured, which is not the case in the LA-free closed conformation, where regions including the RBM were disordered. In the presence of LA, the RBM is tucked away between the RBDs and is inaccessible for ACE2 binding (Toelzer *et al.*, 2020 ▸).

Because of the absence of LA in the previously solved cryo-EM structures and the hitherto elusive ‘locked’ conformation of spike glycoprotein, a key question emerged as to whether this LA-stabilized conformation occurs *in vivo* in mammalian cells. Subsequent to the discovery of the pocket (Toelzer *et al.*, 2020 ▸), LA binding was also observed in numerous cryo-EM structures of SARS-CoV-2 spike protein produced in mammalian cells (PDB entries 7jji, 6zp2, 7dwy, 7xu4, 7xu2, 7xu1, 7xu0 and 7x08; Fig. 2 ▸ *c*; Bangaru *et al.*, 2020 ▸; Xiong *et al.*, 2020 ▸; Yan *et al.*, 2021 ▸; Qu *et al.*, 2022 ▸; Ma *et al.*, 2022 ▸), ruling out a putative insect-cell expression artefact as occasionally surmised. In several other cryo-EM structures, EM density corresponding to LA is present but not assigned (PDB entries 6zgi, 6zge, 6xr8 and 7df3; Fig. 2 ▸ *c*; Wrobel *et al.*, 2020 ▸; Cai *et al.*, 2020 ▸; Xu *et al.*, 2021 ▸). In most of the spike-protein structures derived from mammalian cells, the LA-bound spike proteins are present as a small subpopulation in the data set. More recently, LA binding to insect-cell-produced spike protein was confirmed in SARS-CoV-2 spike at 2.27 Å resolution (Fig. 2 ▸ *c*; PDB entries 7qur and 7qus; Buchanan *et al.*, 2022 ▸). Furthermore, a spike protein (produced in insect cells) from a pangolin-infecting coronavirus was shown to contain LA in the pocket (PDB entry 7cn8; Zhang *et al.*, 2021 ▸), demonstrating that the LA pocket is present in betacoronaviruses that infect nonhuman species. It is evident that a higher population of LA-bound locked spike is found when spike is produced in insect cells compared with that produced in mammalian systems. The lower occupancy of the locked state of spike produced in mammalian expression systems is consistent with a low intracellular LA concentration in mammalian cells. Taken together, the data indicate that the spike protein monitors how much LA is available in its environment and adopts the locked LA-bound structure accordingly.

There are at least two possible explanations for the observed discrepancy in the cryo-EM structures showing high LA occupancy in spike from insect cells and low LA content in spike structures from mammalian cells. Firstly, LA is not present in the minimal media used for mammalian cell culture, whereas human blood serum contains free fatty acids (FFAs), including LA. The media used for insect-cell culture contain FFAs as additives, thus mimicking the host environment. Secondly, the transfection of a plasmid encoding spike protein into mammalian cells does not faithfully recapitulate SARS-CoV-2 viral infection. The latter causes extensive membrane and lipidome remodelling and cPLA_2_ activation (see below), resulting in increased release of LA from the membranes and increased intracellular LA concentration (Müller *et al.*, 2018 ▸; Yan *et al.*, 2019 ▸; Pungerčar *et al.*, 2021 ▸). Baculovirus infection of insect cells to produce spike protein is accompanied by lipid metabolome remodelling by the virus (Nagamine *et al.*, 2019 ▸), which is also likely to contribute to a higher intracellular concentration of FFAs, including LA, in infected insect cells.

### 2.1. Evolutionary conservation of LA binding to the hydrophobic pocket in spike

Sequence alignments of spike proteins of the other human betacoronaviruses SARS-CoV, MERS-CoV, HKU1-hCoV and OC43-hCoV indicated that the hydrophobic pocket in the RBD (named the B domain in HKU1-hCoV and OC43-hCoV) is conserved (Toelzer *et al.*, 2020 ▸). Importantly, the pocket is also conserved in all SARS-CoV-2 variants of concern (VOCs) to date, including the current VOC Omicron (Toelzer *et al.*, 2022 ▸). Using surface plasmon resonance, betacoronavirus spike RBD proteins were tested for LA binding. It was shown that the highly pathogenic SARS-CoV, MERS-CoV and SARS-CoV-2 VOCs indeed bind LA with similar affinity as wild-type SARS-CoV-2 (Wuhan). In marked contrast, HKU1-hCoV, which causes a common cold, has a hydrophobic pocket in the spike RBD but cannot bind LA (Toelzer *et al.*, 2022 ▸). The crystal structure of HKU1-hCoV spike RBD indicates that the entrance to the hydrophobic pocket is obstructed by a glutamate residue (Ou *et al.*, 2017 ▸). Notably, mutation of the glutamate residue to an alanine at the pocket entrance restores LA binding (Toelzer *et al.*, 2022 ▸). Cryo-EM structures of OC43-hCoV spike also showed a hydrophobic pocket within the B domain, but it appears to be spatially too restricted to allow LA binding (Tortorici *et al.*, 2019 ▸; Bangaru *et al.*, 2022 ▸). Moreover, spike protein of pangolin-infecting coronavirus was shown to bind LA (Zhang *et al.*, 2021 ▸), confirming that the fatty-acid pocket in spike is conserved in betacoronaviruses infecting other species.

Targeted molecular-dynamics (MD) simulations were performed to assess the relative stability of the closed and LA-bound locked spike proteins (Gupta *et al.*, 2022 ▸). A harmonic force constant of 0.2 kJ mol^−1^ nm^−1^ was applied to raise a single RBD from the closed conformation. In 98% of the 50 simulations (10 ns each) application of the force resulted in an opening of the closed spike in the absence of LA. In contrast, only 14% of the LA-bound spikes opened on the application of the same force. This demonstrates that LA binding stabilizes the locked state of spike, increasing the energy required to raise a single RBD and reach an open conformation (Gupta *et al.*, 2022 ▸). Non-equilibrium MD simulations showed an allosteric connection between the LA-binding pocket and the fusion peptide-proximal region, the region preceding the S2′ site and the furin-cleavage site, as shown by conformational rearrangements in these regions after LA removal from spike (Gupta *et al.*, 2022 ▸; Oliveira, Shoemark, Avila Ibarra *et al.*, 2022 ▸).

In addition to the LA-binding pocket itself, the RBD–RBD interface was computationally analysed. The RBD of VOC B1.617.2-AY1 (Delta plus) spike was found to contain a lysine-to-asparagine mutation (K417N) leading to loss of a positive charge at the RBD–RBD interface. This may affect the intersubunit LA–RBD interaction and weaken the stabilization effect of LA in this VOC spike (Shoemark *et al.*, 2022 ▸). Moreover, in the RBD of the Omicron variant BA.2, the arginine (Arg408 in Wuhan spike; Fig. 2 ▸ *b*) which coordinates the carboxy headgroup of LA (Fig. 2 ▸) is mutated to a serine residue in addition to the K417N mutation (as in Delta plus spike RBD). The R408S mutation is suggested to reduce the affinity of LA for the pocket, and both mutations are likely to affect the RBD–RBD interactions, leading to reduced stability of the locked spike conformation (Shoemark *et al.*, 2022 ▸). Consistently, in equilibrium MD simulations, the RBD–RBD interface is less tightly packed in the Omicron BA.2 VOC. However, despite this interface destabilization, which is suggested to facilitate opening of the RBDs, none of the LA molecules dissociated from the Omicron spike LA pocket during 200 ns MD simulations (Shoemark *et al.*, 2022 ▸). Vice versa, binding of LA is also suggested to reduce the frequency of opening of spike RBDs in Omicron spike. Interestingly, dynamic non-equilibrium MD simulations indicate that removal of LA from the LA pocket of spike perturbs the conserved allosteric interaction network with the RBM and with the furin-cleavage site more in Omicron spike than in wild-type (Wuhan) spike and other VOCs (Oliveira, Shoemark, Davidson *et al.*, 2022 ▸). In contrast, the fusion peptide-proximal region of Omicron spike is affected less by LA removal (Oliveira, Shoemark, Davidson *et al.*, 2022 ▸). In summary, it appears that the mutations in the RBD of VOC spikes affect how efficiently LA binds and how structural changes in the RBD are propagated to other functional sites, but they do not abolish LA binding to the pocket in spike.

### 2.2. Does LA binding provide a selective advantage to the virus?

The observed remarkable evolutionary conservation of the pocket and of LA binding raises the question of the physiological role of LA binding and the selective advantage for the virus. In fact, the hydrophobic pocket has remained virtually unchanged for at least 20 years since SARS-CoV emerged, indicating that this is an important functional feature of the spike proteins of these highly pathogenic human betacoronaviruses.

On the molecular level, it appears that spike proteins need to maintain a fine balance between stability and infectivity, which can be achieved by switching from closed or locked to open conformations (Berger & Schaffitzel, 2020 ▸), with the open state being less stable and prone to S2′ proteolytic digestion and dissociation of the S1 subunits. The LA-bound locked spike stabilizes the spike trimer and prevents it from binding the ACE2 receptor, as shown *in vitro* using surface plasmon resonance assays (Toelzer *et al.*, 2020 ▸). Importantly, inhibition of viral infection and replication was observed with live SARS-CoV-2 virus, where the addition of 50 µ*M* LA and remdesivir to the medium had a significantly stronger effect than remdesivir alone (Toelzer *et al.*, 2020 ▸). Remdesivir was the first approved SARS-CoV-2 drug that inhibits the viral RNA-dependent RNA polymerase. However, remdesivir has significant side effects at the doses required (Mohammad Zadeh *et al.*, 2021 ▸). By interfering with viral infection, LA can be used to substantially decrease the effective dose required for remdesivir to block viral replication, potentially reducing side effects (Toelzer *et al.*, 2020 ▸). A direct effect of LA on spike–ACE2 receptor binding on the host cell surface has been observed with synthetic SARS-CoV-2 mini-virions displaying functional spike protein (Staufer *et al.*, 2022 ▸). SARS-CoV-2 mini-virions consist of vesicles identical in size and lipid composition to SARS-CoV-2 virions reproducing the viral membrane envelope. Mini-virions can readily be decorated with recombinant spike proteins at the same density as in SARS-CoV-2 (Staufer *et al.*, 2022 ▸). When mini-virions with spike were treated with the unsaturated free fatty acids LA, oleic acid (OA) or arachidonic acid (AA) (1 µ*M* each), significantly reduced attachment to human cells was observed compared with mini-virions with apo spike devoid of LA. Importantly, saturated fatty acids such as palmitic acid (PA) did not show this inhibitory effect, and the mini-virions bound with similar efficiency as mini-virions with apo spike (Staufer *et al.*, 2022 ▸). Less efficient ACE2 receptor binding by the LA-bound spike is expected to impact all subsequent steps: the proteolytic S2′ cleavage, conformational rearrangement and membrane-fusion steps, leading to less efficient host cell infection (Staufer *et al.*, 2022 ▸).

**Figure 3:**
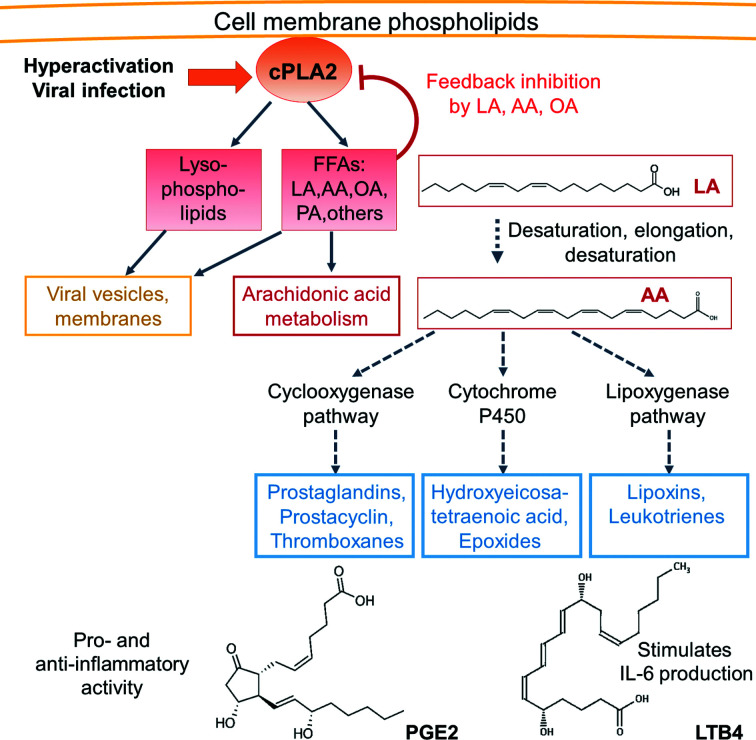
cPLA_2_ hyperactivation and the arachidonic acid metabolism. During viral infection, cPLA_2_s are activated, releasing FFAs and lysophospholipids from the membrane to generate viral compartments and virions. Free LA inhibits cPLA_2_ and is metabolized into AA (chemical structures shown). AA is converted into diverse eicosanoids via three main pathways: cyclooxygenases, P450 and lipoxygenases. The resulting products, including prostaglandins (PG) and leukotrienes (LT), have important roles in the regulation of immune response (cytokine production), inflammation and beyond.

Inside the host cell, SARS-CoV-2 infection causes activation of cytosolic phospholipase A_2_ (cPLA_2_), an enzyme target for antiviral drug development (Müller *et al.*, 2018 ▸; Fig. 3 ▸). Activated cPLA_2_ releases FFAs including LA, AA, OA and PA from the plasma membrane and supports the extensive membrane remodelling leading to the formation of the intra­cellular double-membrane vesicles and replicative organ­elles required for viral replication (Müller *et al.*, 2018 ▸; Yan *et al.*, 2019 ▸; Pungerčar *et al.*, 2021 ▸). In fact, cellular lipid metabolism is severely perturbed in coronavirus-infected cells, and virus replication critically depends on the enzymes involved in lipid metabolism, including cPLA_2_ (Müller *et al.*, 2018 ▸). cPLA_2_ has been shown to be regulated by polyunsaturated fatty acids (PUFAs) in a negative-feedback mechanism (Fig. 3 ▸). Consistently, LA, AA and OA are competitive inhibitors of cPLA_2_ *in vitro* (Ballou & Cheung, 1985 ▸; Toelzer *et al.*, 2022 ▸). Moreover, LA treatment of infected cells has been shown to inhibit the replication of MERS-CoV, of alphacoronavirus 229E (Yan *et al.*, 2019 ▸) and of SARS-CoV-2 (Toelzer *et al.*, 2022 ▸). Consistently, LA-treated infected cells contained fewer SARS-CoV-2 virions and the virions produced were deformed in size and shape (Toelzer *et al.*, 2022 ▸).

During and after egress from the host cell, LA-locked spike presumably allows SARS-CoV-2 to travel further in the serum because spike is in a non-infectious conformation. LA is likely to impede the opening of the RBDs (Oliveira, Shoemark, Avila Ibarra *et al.*, 2022 ▸). Subsequently, LA only slowly dissociates from spike, as shown by the fact that LA-bound spike did not lose most of the bound LA during the protein-purification procedure and cryo-EM grid preparation (Toelzer *et al.*, 2020 ▸, 2022 ▸; Gupta *et al.*, 2022 ▸). Moreover, in the closed/ locked state, S1 shields the S2 fusion machinery and prevents premature cleavage of S2′ by host cell proteases, for example TMPRSS2 (Hoffmann, Kleine-Weber, Schroeder *et al.*, 2020 ▸). Consistent with this, *in situ* cryo-tomograms of extracellular SARS-CoV-2 virions from infected Vero E6 and Calu cells, which were grown in minimal media in the absence of FFAs, showed a significant portion of spike proteins in the post-fusion conformation (Ke *et al.*, 2020 ▸; Klein *et al.*, 2020 ▸), indicating that premature cleavage of spike occurred, leading to rearrangement into the post-fusion state without docking of SARS-CoV-2 to a new host cell. These post-fusion spike proteins have not been observed in virus isolates from patients, where the virus is decorated with the intact spike proteins, indicating that the cell culture does not faithfully reproduce the situation *in vivo*.

By binding LA, spike adopts a locked conformation, decreases the exposure of immunogenic sites in the RBD and couples the immunogenicity of spike to LA levels in the serum of the host (Staufer *et al.*, 2022 ▸). Staufer and coworkers calculated the accessible surface area of open and LA-bound locked spike conformations to be ∼1.7 to 1 for binding sites of neutralizing IgG antibodies, providing evidence that key neutralization sites, including the RBM, are only exposed in the open spike conformation (Staufer *et al.*, 2022 ▸). In contrast, LA-bound spike can temporarily escape the immune response by hiding immunogenic epitopes.

The N-terminal domain (NTD) of SARS-CoV-2 spike (Wuhan) has been shown to bind the heme metabolites bilirubin and biliverdin (Rosa *et al.*, 2021 ▸). The binding of biliverdin to spike interfered with the binding of a subset of neutralizing antibodies. It was suggested that this could present a novel mechanism of immune evasion by allostery (Rosa *et al.*, 2021 ▸). Similarly, early pandemic spike NTD was shown to bind sialoside sugars (Buchanan *et al.*, 2022 ▸). Such sugar motifs were identified in N-linked glycoproteins in the human lung, and spike binding to these sugars is suggested to have contributed to disease severity (Buchanan *et al.*, 2022 ▸). Interestingly, the binding sites for heme and sialoside sugar ligands have been mutated in subsequent SARS-CoV-2 variants (Rosa *et al.*, 2021 ▸; Buchanan *et al.*, 2022 ▸), whereas the LA-binding pocket remained conserved in the current variant of concern Omicron (Toelzer *et al.*, 2022 ▸).

## 3. Lipids and fatty acids are generic regulators of viral infection

**Figure 4:**
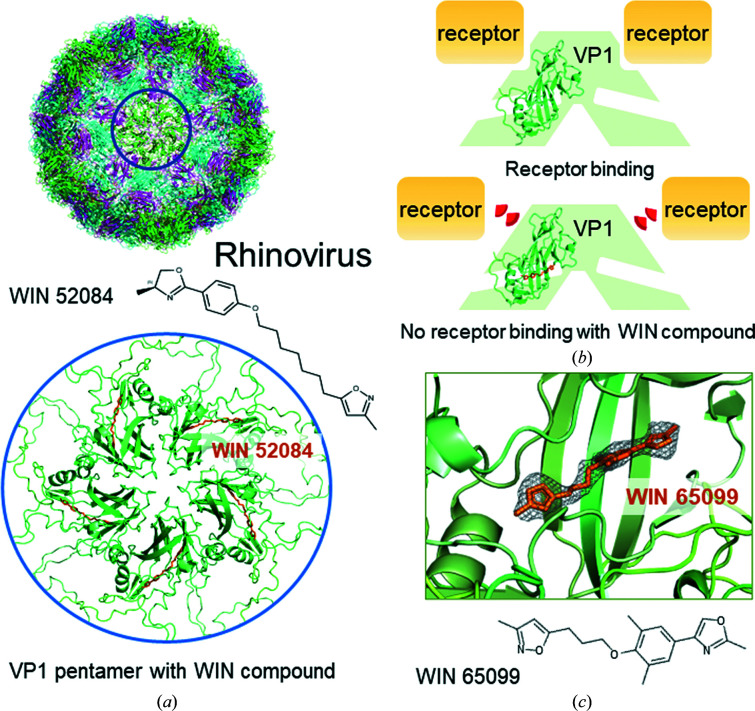
WIN compounds inhibit VP1 receptor binding. (*a*) Structure of the human rhinovirus HRV14 capsid in complex with the antiviral compound WIN 52084 comprising the coat protein VP1 (green), VP2 (cyan), VP3 (magenta) and VP4 (yellow). A pocket factor, most likely a fatty acid, bound within a hydrophobic pocket of VP1 inspired the synthesis of the WIN compounds. The blue circle highlights a VP1 pentamer, shown in a close-up view (below) bound with WIN 52084 (orange, chemical structure shown above; PDB entry 1rud; Hadfield *et al.*, 1995 ▸). (*b*) Schematic showing receptor binding to a cleft in the VP1 pentamer in the absence of a bound WIN compound or pocket factor. Below: WIN compounds bind tightly to the pocket and induce a conformation that is incompatible with receptor binding to VP1. (*c*) Zoom into the hydrophobic pocket of human rhinovirus HRV16 VP1 occupied by WIN 65099 (orange and mesh, chemical structure shown below; PDB entry 1qjy; Hadfield *et al.*, 1999 ▸).

Interestingly, fatty-acid and lipid binding has also been reported for other viral surface proteins. Here, we highlight examples from picornaviruses, Zika virus and alphaviruses. Several picornaviruses contain a hydrophobic pocket in their viral capsid protein VP1, including human enterovirus 71, which causes hand, foot and mouth disease (Plevka *et al.*, 2013 ▸), and rhinovirus 14, which causes upper respiratory illness (Fig. 4 ▸; Hadfield *et al.*, 1995 ▸). A canyon-like depression is found in the virus capsid protein, which is where host cell receptors bind (Plevka *et al.*, 2013 ▸; Figs. 4 ▸ *a* and 4 ▸ *b*). Receptor binding to the canyon displaces a hydrophobic ‘pocket factor’ from a pocket in VP1 below the canyon (Fig. 4 ▸ *b*). This ‘pocket factor’ in the VP1 pocket has been suggested to stabilize picornavirus assembly. Receptor recognition and concomitant loss of the ‘pocket factor’ destabilizes the capsid and helps to release the picornavirus RNA genome during infection (Rossmann *et al.*, 2002 ▸). Chemical analyses of virions from bovine enterovirus indicate that the ‘pocket factor’ is a mixture of lipids with a prevalence of palmitic acid and myristic acid (Smyth *et al.*, 2003 ▸). Small-molecule inhibitors, named WIN compounds, have been developed as antivirals against rhinoviruses, poliovirus and enterovirus infection. These WIN compounds bind with high affinity to the hydrophobic pocket, stabilize the virus capsid and interfere with receptor binding and infection (Fig. 4 ▸ *c*; Hadfield *et al.*, 1995 ▸, 1999 ▸; Smith *et al.*, 1986 ▸; Filman *et al.*, 1989 ▸; Plevka *et al.*, 2013 ▸). The WIN compound vapendavir has been tested in Phase IIb clinical trials for antirhinovirus activity and indeed showed a reduction of viral load; development is ongoing (Lanko *et al.*, 2021 ▸).

Zika virus is a positive-strand RNA virus. The RNA is packaged in a host-derived lipid bilayer and is covered by membrane-anchored envelope protein E, which is responsible for receptor binding and cell entry, as well as membrane protein M (DiNunno *et al.*, 2020 ▸). A recent cryo-EM structure showed that the M and E proteins form a heterodimer. In the structure, the two proteins form a hydrophobic cleft binding a lipid close to the inner membrane leaflet. Moreover, protein E forms a hydrophobic pocket which is positioned above the outer membrane leaflet and is likely to also be occupied by a lipid (DiNunno *et al.*, 2020 ▸). Subsequently, it was shown that mutation of the hydrophobic residues lining the pocket in protein E was detrimental to Zika virus. Consistently, the bound lipids are suggested to stabilize the viral E protein or support its maturation (DiNunno *et al.*, 2020 ▸). Alignment of E protein sequences from different flaviviruses indicates that the residues lining the lipid-binding pocket are highly conserved and lipid binding could be essential for all flaviviruses.

Sindbis virus, which belongs to the family of alphaviruses, has also been shown to contain a hydrophobic pocket in its E2 protein which is responsible for host cell receptor binding (Chen *et al.*, 2018 ▸). The pocket is occupied by an unknown hydrophobic ‘pocket factor’ and is located near the viral membrane in the cryo-EM structure. Sequence conservation of the hydrophobic residues lining the pocket suggests that the pocket is also present in other alphaviruses. In fact, a similar EM density is observed in the hydrophobic pocket of E2 of Venezuelan equine encephalitis virus (Zhang *et al.*, 2011 ▸). It is hypothesized that the hydrophobic ligand in the pocket is a phospholipid tail that stabilizes the E2 protein, facilitates virus assembly and is likely to be released during viral entry (Chen *et al.*, 2018 ▸).

Taken together, a picture emerges in which FFAs and lipids stabilize a non-infectious form of viral surface proteins, and they need to be dissociated before or during receptor binding to allow infection of the host cell.

### 3.1. The hydrophobic pocket in spike is druggable

The findings above suggest that the pocket in SARS-CoV-2 spike may be a drug target for inhibition of infection. Using docking, MD simulations and a library of FDA-approved drugs, ligand binding to the hydrophobic pocket in SARS-CoV-2 spike was investigated (Shoemark *et al.*, 2021 ▸). Based on the virtual screening experiments, the steroid dexamethasone and the fat-soluble vitamins A, D and K as well as retinoids were suggested to bind to the hydrophobic pocket and stabilize the closed/locked conformation of spike (Shoemark *et al.*, 2021 ▸). Of the retinoids, all-*trans* retinoic acid (ATRA) and vitamin A were shown to have antiviral activity against SARS-CoV-2. A recent cryo-EM structure reveals that ATRA indeed binds to the hydrophobic pocket of SARS-CoV-2 spike protein (Tong *et al.*, 2022 ▸). Similar to LA binding, ATRA binds with its hydrophobic tail to one RBD and interacts with Arg408 and Gln409 of the adjacent RBD. Thereby, ATRA stabilizes a closed/locked spike conformation which only inefficiently interacts with the ACE2 receptor (Tong *et al.*, 2022 ▸). However, the affinity of ATRA and vitamin A for spike is in the micromolar range (3 µ*M* for ATRA; Tong *et al.*, 2022 ▸), which is significantly lower than the affinity of LA for one RBD alone (*K*_d_ = ∼41 n*M*). Importantly, ATRA inhibits the entry of SARS-CoV and MERS-CoV pseudoviruses into human cells, confirming the conservation of the hydrophobic pocket (Tong *et al.*, 2022 ▸).

### 3.2. LA regulates immune response and inflammation

The human body cannot synthesize LA; LA is a vitamin that needs to be taken up in the diet. It is remarkable in this context that SARS-CoV-2 spike binds LA with astonishing specificity. In the human body, LA is converted to arachidonic acid (AA) by desaturation and elongation (Hanna & Hafez, 2018 ▸). LA and AA are further metabolized via oxidation to eicosanoids, including prostaglandins, leukotrienes and short-lived lipoxins (Fig. 3 ▸). Eicosanoids are key lipid signalling molecules that act on cells via G-protein coupled receptors (GPCRs), regulating acute inflammation and important aspects of immunity, including cytokine production, antibody formation and antigen presentation (Harizi *et al.*, 2008 ▸; Zaman *et al.*, 2010 ▸). Prostaglandins can activate or downregulate inflammation depending on the cellular context (Goodwin *et al.*, 2015 ▸). All eicosanoids, their receptors and cytokines are known to cooperate to modulate cell homeostasis and inflammation (Harizi *et al.*, 2008 ▸). LA itself is detected by the free fatty-acid receptor GPR40 (also named FFAR1), which is also a GPCR (Zhou *et al.*, 2012 ▸). GPR40-mediated FFA signalling is a powerful mediator of inflammation in human tissues and in animal models (Kimura *et al.*, 2020 ▸).

Under physiological conditions, the levels of unsaturated FFA are maintained below 0.1 µ*M* (Richieri & Kleinfeld, 1995 ▸; Brash, 2001 ▸). During COVID-19 and lung inflammation, however, fatty-acid levels can temporarily increase up to 1.1 µ*M* in a so-called fatty acid–lipid storm (Yan *et al.*, 2019 ▸; Archambault *et al.*, 2021 ▸). Consistently, in severe cases of COVID-19 LA levels were significantly increased in saliva (Saheb Sharif-Askari *et al.*, 2022 ▸). Metabolomics studies also found increased levels of polyunsaturated fatty acids (PUFAs) and lipids in the plasma of patients with COVID-19 compared with healthy individuals (Occelli *et al.*, 2022 ▸; Song *et al.*, 2020 ▸; Schwarz *et al.*, 2021 ▸; Wu *et al.*, 2020 ▸). The hyperactivation of cPLA_2_s releasing long-chain PUFAs from host cell membranes has been implicated in severe COVID-19 (Barberis *et al.*, 2020 ▸; Casari *et al.*, 2021 ▸; Fig. 3 ▸). The observed strong increases in lipid and FFA levels (dyslipidemia) in the serum of severe COVID-19 patients have been suggested to be linked to multi-organ deterioration, cytokine storm, hypercoagulation and pneumonia (Shen *et al.*, 2020 ▸; Su *et al.*, 2020 ▸).

The dysregulation of FFA levels during COVID-19 is suggested to result from cPLA_2_ activation and lipid metabolome remodelling, which are both common elements of viral infection (Goodwin *et al.*, 2015 ▸; Yan *et al.*, 2019 ▸). Host cell lipid metabolome remodelling by RNA viruses is essential to generate replicative organelles, organize viral assembly and modulate the inflammatory environment. Lipid remodelling alters at least three separate processes in cells (Yan *et al.*, 2019 ▸; Goodwin *et al.*, 2015 ▸). (i) Cell signalling is changed due to altered eicosanoid levels (see above). (ii) The energy homeostasis is altered by rewiring of metabolic pathways during viral infection (Goodwin *et al.*, 2015 ▸). For instance, transcriptome analyses of lung epithelial cells revealed a substantial up­regulation of cholesterol and fatty-acid biosynthesis, accompanied by upregulation of glycolysis and dysregulation of the citric acid cycle, induced by SARS-CoV-2 proteins (Ehrlich *et al.*, 2020 ▸). Consistently, lipids have been shown to accumulate in SARS-CoV-2-infected cells (Ehrlich *et al.*, 2020 ▸). (iii) The fluidity and elasticity of biological membranes are affected, for example, via changes in the ratio of unsaturated and saturated fatty acids in phospholipids. High levels of saturated fatty acids in phospholipids are known to rigidify the membrane. Accordingly, the composition of fatty acids in the phospholipid bilayer is a key element in maintaining surface tension in lungs. Differences in fatty-acid diet have been reported to affect the lipid composition in membranes and eicosanoid production (Leaf & Weber, 1988 ▸; Schwartz, 2000 ▸). Consistently, alteration of LA, AA and metabolite lipid composition is observed in acute respiratory distress syndrome and severe pneumonia (Schmidt *et al.*, 2001 ▸), both of which are key symptoms of SARS-CoV-2 infection.

## 4. Conclusion

LA binding to an evolutionarily conserved hydrophobic pocket of the RBD of SARS-CoV-2 spike protein was discovered in the spike cryo-EM structure and substantiated by LC-MS-MS. LA impacts viral infection and disease progression by several mechanisms. LA stabilizes spike protein in a non-infectious locked conformation, avoiding premature S2′ cleavage and dissociation of the S1 subunit of spike. Important immunogenic epitopes in the RBD of spike, including the RBM, evade immune recognition in the LA-stabilized locked conformation. In the human host cell, LA binding to spike and viral membranes contributes to hyperactivation of cPLA_2_ and lipid remodelling, allowing the formation of viral replication compartments. LA release from the membranes contributes to the later-stage fatty acid–lipid storm caused by pro-inflammatory eicosanoids. Release of PUFAs, including LA, from the membrane by cPLA_2_ changes the fluidity of the cellular membranes and contributes to pulmonary and cardiovascular disease. Taken together, this suggests that early-stage supplementation of LA, *i.e.* while the viral infection is localized in the upper airways, may be beneficial to interfere early with SARS-CoV-2 infection, replication and viral transmission. Current insights into the mechanism of SARS-CoV-2 infection further provide a rationale for the development of intranasal, pulmonary or oropharyngeal drug-delivery systems for self-administration, targeting mucosal epithelial cells with high ACE2 concentration. Given worldwide persisting SARS-CoV-2 infections and the conservation of the pocket in spike protein, LA delivery could present a novel and sustainable strategy for early COVID-19 treatment and prophylaxis.
